# Occupational exposures in the operating room: Are surgeons well-equipped?

**DOI:** 10.1371/journal.pone.0253785

**Published:** 2021-07-02

**Authors:** Wilmina N. Landford, Ledibabari M. Ngaage, Erica Lee, Yvonne Rasko, Robin Yang, Sheri Slezak, Richard Redett

**Affiliations:** 1 Department of Plastic and Reconstructive Surgery, Johns Hopkins University School of Medicine, Baltimore, Maryland, United States of America; 2 Division of Plastic and Reconstructive Surgery, Department of Surgery, University of Maryland Medical Center, Baltimore, Maryland, United States of America; Indiana University, UNITED STATES

## Abstract

**Background:**

Occupational health hazards are ubiquitously found in the operating room, guaranteeing an inevitable risk of exposure to the surgeon. Although provisions on occupational health and safety in healthcare exist, they do not address non-traditional hazards found in the operating room. In order to determine whether surgeons or trainees receive any form of occupational health training, we examine the associations between occupational health training and exposure rate.

**Study design:**

A cross-sectional survey was distributed. Respondent characteristics included academic level, race/ethnicity, and gender. The survey evaluated seven surgical disciplines and 13 occupational hazards. Multivariable logistic regression was used to examine the association between academic level, surgical specialty, and exposure rate.

**Results:**

Our cohort of 183 respondents (33.1% response rate) consisted of attendings (n = 72, 39.3%) and trainees (n = 111, 60.7%). Surgical trainees were less likely to have been trained in cytotoxic drugs (OR 0.22, *p*<0.001), methylmethacrylate (OR 0.15, *p*<0.001), patient lifting (OR 0.43, *p* = 0.009), radiation (OR 0.40, *p* = 0.007), and surgical smoke (OR 0.41, *p* = 0.041) than attending surgeons. Additionally, trainees were more likely to experience frequent exposure to bloodborne pathogens (OR 5.26, *p*<0.001), methylmethacrylate (OR 2.86, *p*<0.001), cytotoxic drugs (OR 3.03, *p*<0.001), and formaldehyde (2.08, *p* = 0.011), to name a few.

**Conclusion:**

Although surgeon safety is not a domain in residency training, standardized efforts to educate and change the culture of safety in residency programs is warranted. Our study demonstrates a disparity between trainees and attendings with a recommendation to provide formal training to trainees independent of their anticipated risk of exposure.

## Introduction

Surgeons are frequently exposed to a host of occupational hazards that all pose an established risk to their health and safety. In the operating room (OR), the ubiquity of these hazards guarantees inevitable exposure to blood-borne pathogens for approximately 5.6 million healthcare workers, an average of 5-50mrem/case, 10-350mrem/month, or 2000-3000mrem/year of radiation depending on the surgical specialty, and biological by-products from surgical smoke [1–4]. Accordingly, the collaborative nature of the surgical field places surgeons at risk of exposure to hazards not always specific to their own specialty. Vascular surgeons, Orthopedic surgeons, and Plastic surgeons working together on a complex operation may use intraoperative technologies that emit radiation or produce vibratory sound above recommended levels that may not be typical within their own respective specialties [3, 5]. While efforts have been made to improve OR safety through mandated occupational safety trainings, no policies or trainings exist on non-traditional hazards that address the chemical or biological materials emitted from laser or surgical smoke, anesthetic gases, or ergonomics, to name a few [1, 2].

Institutional practices and personal behaviors play a significant role in the implementation of occupational health and safety provisions. Current literature implies that underreporting of workplace injuries is prevalent and multifactorial in the surgical field [6–8]. A unique challenge to instilling policies is the lack of established exposure limits for several of the hazards as expert agency guidelines are either lacking or outdated. Several studies have demonstrated an increased incidence of malignancy, infertility, and shortened lifespan following prolonged and repeated exposure to common hazards in the OR [9–14]. However, these conclusions are based on a small number of studies and low-level evidence. Moreover, the literature is near void on the consequences of prolonged exposure over a surgeon’s career. Consequently, physicians may remain ignorant on the potential risks. Therefore, early awareness and education is imperative in reducing the economic, personal, and social implications of these non-traditional hazards.

The purpose of this study was to elucidate the extent to which surgeons are trained in OR hazards and assess the self-reported exposure rate across surgical specialties and academic level.

## Methods

### Study population & recruitment

The Institutional Review Board (IRB) at the Johns Hopkins University School of Medicine approved this study and deemed it exempt. A cross-sectional electronic questionnaire, Qualtrics (Qualtrics, version 1-0-0, Provo, CT) online survey and research tool, was distributed to surgical attendings (*n* = 72 (39.3%)), fellows (*n* = 11 (6%)), and residents (*n* = 100 (54.6%)) from June through August 2019 at Johns Hopkins Hospital and affiliated hospitals. No compensation was offered for completion of the survey. Recruitment for individual respondents was through email using the surgery department lists. Participants were invited to complete an online questionnaire through which informed consent was obtained. Incomplete surveys were avoided through completion control implemented in the online tool. Non-respondents were automatically reminded to complete the survey after 4 weeks through the Qualtrics platform. A total of three reminders were sent to nonrespondents. The non-respondent population was characterized by comparing demographic information of the entire emailed cohort to that of respondents.

### Measures

Occupational hazards were selected following three round table discussions with a multidisciplinary group of surgeons at all academic levels. The survey instrument was revised from 28-items to 21-items based on the review, rewritten for clarity, and electronic survey logic was added. The revised questionnaire was pilot tested by a group of residents and attendings that represented the cohort of interested consisting of surgeons and trainees from the same institution. The multidisciplinary team was not included in the final survey study. Feedback was incorporated before the survey tool was finalized. This study followed the American Association for Public Opinion Research (AAPOR) disclosure checklist. All questions were multiple choice and included demographic information, current academic level, and surgical specialty. Fellows and residents were categorized as trainees. Surgical disciplines included General, Otolaryngology, Neurosurgery, Orthopedics, Plastic & Reconstructive surgery, Urology, and OBGYN. General surgery included Cardiac, Pediatric, and Vascular surgery.

The survey assessed four key areas: (i) whether the participants had ever received formal occupational hazard training, (ii) frequency of training, (iii) adequacy of training, and (iv) the frequency of exposure to occupational hazards within the last year. The frequency of training was reported as never received, received once during career, received yearly, or received monthly. Adequacy of training was evaluated on a five-point Likert scale where 1 = unacceptable and 5 = excellent.

The survey evaluated 13 occupational hazards: bloodborne pathogens, surgical smoke, ergonomics, radiation, sharp injuries, inhalation exposure to methylmethacrylate, cytotoxic drugs, formaldehyde, patient lifting, prolonged standing (more than 3 hours), surgical hand scrub, surgical noise (anesthesia machines, monitors, vibratory devices, suctioning, music), and anesthetic gases. Bloodborne pathogens were described as blood products, patient fluid products released during tissue debridement, and intraoperative blood products. Surgical smoke pertained to surgical plume released from cautery, cautery-like devices, or laser surgeries. Radiation sources included fluoroscopic intraoperative imaging (i.e. C-arm, mini C-arm) and plain radiographs. Ergonomics referred to intraoperative equipment use and use of loupes. Sharp injuries pertained to both needlestick and sharp injuries. Cytotoxic drugs included HIPEC, PIPAC, and mitomycin.

### Statistical analysis

Data was compiled in Qualtrics and then converted to a Microsoft Excel spreadsheet (Microsoft 2016, Redmond, Washington) and analyzed using the Statistical Package for the Social Sciences (SPSS, Version 26 IBM Corp, 2019. IBM SPSS Statistics for Windows, Armonk, NY: IBM Corp.). Descriptive analyses were performed to examine the individual effects of demographic, academic level, and surgical specialty. Surveys a with <90% item response rate and subgroups with fewer than 5 respondents were excluded from analysis. A total of 3 surveys were excluded following this exclusion criteria. Due to the low number of respondents who received training in any occupational hazard on a monthly basis (n = 2), monthly training was merged with the group that received annual training and called “annual or more frequent”. Exposure frequency was converted to a numeric ordinal scale for the purpose of analysis; 0 –never exposed, 1 –exposed once, 2 –yearly, 3 –monthly, 4 –weekly, 5 –few times per week, and 6 –daily. We reported the median and interquartile range for ordinal data. The Mann Whitney-U and Kruskal Wallis test were used to assess for significant differences in satisfaction, as appropriate. We calculated odds ratios (OR) and 95% confidence intervals (CI) to evaluate associations between training received on occupational hazards and the variable of interest (academic level and surgical specialty). Ordinal regression analysis was used to assess associations between exposure frequency and the variable of interest (academic level, surgical specialty, and receipt of occupational hazard training). Statistical significance was defined as a two-tailed value of *p*≤0.05.

## Results

A total 183 of 553 respondents (33.1% response rate) participated in the survey. Differences between the respondent and non-respondent populations are summarized in S1 Table. Among respondents, over half included surgical trainees (*n* = 111, 60.7%) and the rest were attendings. Respondents were categorized into seven surgical specialties, with a majority from General Surgery (30.6%). Ethnic representation demonstrated a predominantly Caucasian cohort. A complete summary of all demographic and training level characteristics can be found in S1 Table.

### Occupational hazard training

Respondents were asked to indicate whether they had received any form of training or educational material on 13 occupational OR hazards. Nearly all respondents reported receiving some form of training or material on bloodborne pathogens (96.2%) and needlestick/sharp injuries (96.2%). Two-thirds of respondents were trained in radiation (64.5%) and methylmethacrylate (64.5%) but very few respondents received formal training on surgical noise (12.6%) or prolonged standing (7.1%) (S2 Table). When respondents were stratified by academic level and surgical specialty, significant differences were noted in the receipt of chemical and physical occupational hazard training (Tables 1–3).

**Table 1:** Receipt and frequency of formal training for biological occupational hazards stratified by academic level and surgical specialty.

Characteristic	Receipt of formal training			Annual (or more frequent) training		
	No. (%)	OR (95% CI)	*p*-value	No. (%)	OR (95% CI)	*p*-value
**Bloodborne pathogens**						
*Academic level*						
Attending	69 (95.8%)	1.0		42 (60.9%)	1.0	
Resident/fellow	107 (96.4%)	1.16 (0.25–5.26)	0.846	62 (57.9%)	0.88 (0.48–1.64)	0.700
*Surgical specialty*						
General	52 (92.9%)	1.0		30 (57.7%)	1.0	
Neurological	11 (100%)	0.93 (0.86–1.00)	0.361	5 (45.5%)	0.61 (0.17–2.26)	0.458
OBGYN	19 (100%)	0.93 (0.86–1.00)	0.231	11 (57.9%)	1.01 (0.35–2.92)	0.988
Orthopedic	16 (100%)	0.93 (0.86–1.00)	0.271	8 (50.0%)	0.73 (0.24–2.26)	0.588
Otolaryngology	19 (100%)	0.93 (0.86–1.00)	0.231	12 (63.2%)	1.26 (0.426–3.71)	0.678
Plastic	50 (96.2%)	1.92 (0.34–10.97)	0.455	33 (66.7%)	1.42 (0.64–3.18)	0.388
Urology	9 (90.0%)	0.69 (0.69–6.92)	0.753	5 (55.6%)	0.92 (0.22–3.81)	0.905
**Needlestick or Sharps Injury**						
*Academic level*						
Attending	70 (97.2%)	1.0		39 (55.7%)	1.0	
Resident/fellow	106 (95.5%)	0.61 (0.11–3.13)	0.552	62 (58.5%)	1.12 (0.61–2.06)	0.715
*Surgical specialty*						
General	53 (94.6%)	1.0		27 (50.9%)	1.0	
Neurological	11 (100%)	0.95 (0.89–1.01)	0.432	7 (63.6%)	1.69 (0.44–6.44)	0.443
OBGYN	18 (94.7%)	1.02 (0.10–10.43)	0.987	10 (55.6%)	1.20 (0.41–3.53)	0.735
Orthopedic	15 (93.8%)	0.85 (0.08–8.77)	0.891	7 (46.7%)	0.83 (0.27–2.657)	0.770
Otolaryngology	19 (100%)	0.95 (0.89–1.01)	0.303	14 (73.7%)	2.97 (0.85–8.55)	0.086
Plastic	51 (98.1%)	2.89 (0.29–28.67)	0.345	32 (62.7%)	1.62 (0.74–3.547)	0.225
Urology	9 (90.0%)	0.51 (0.05–5.46)	0.571	4 (44.4%)	0.77 (0.19–3.19)	0.718

CI, confidence interval; OBGYN, obstetrics and gynecology; OR, odds ratio

**Table 2:** Receipt and frequency of formal training for chemical occupational hazards stratified by academic level and surgical specialty.

Characteristic	Receipt of formal training			Annual (or more frequent) training		
	No. (%)	OR (95% CI)	*p*-value	No. (%)	OR (95% CI)	*p*-value
**Anesthetic gases**						
*Academic level*						
Attending	25 (34.7%)	1.0		7 (28%)	1.0	
Resident/fellow	24 (21.6%)	0.52 (0.27–1.01)	0.051	7 (29.2%)	1.06 (0.31–3.70)	0.928
*Surgical specialty*						
General	18 (32.1%)	1.0		3 (16.7%)	1.0	
Neurological	5 (45.5%)	1.76 (0.47–6.54)	0.395	3 (60%)	6.50 (0.73–57.83)	0.093
OBGYN	2 (10.5%)	0.25 (0.05–1.19)	0.066	0 (0.0%)	0.83 (0.68–1.03)	0.531
Orthopedic	1 (6.3%)	0.14 (0.17–1.15)	0.067	1 (100%)	0.17 (0.06–0.47)	**0.047**
Otolaryngology	7 (36.8%)	1.23 (0.42–3.66)	0.707	2 (28.6%)	2.00 (0.26–15.62)	0.504
Plastic	14 (26.9%)	0.78 (0.34–1.79)	0.553	5 (35.7%)	2.78 (0.53–14.50)	0.217
Urology	2 (20.0%)	0.53 (0.10–2.74)	0.442	0 (0.0%)	0.83 (0.68–1.025)	0.531
**Cytotoxic drugs**						
*Academic level*						
Attending	27 (37.5%)	1.0		5 (18.5%)	1.0	
Resident/fellow	13 (11.7%)	0.22 (0.10–0.47)	**<0.001**	5 (38.5%)	2.78 (0.63–12.5)	0.172
*Surgical specialty*						
General	13 (23.2%)	1.0		2 (15.4%)	1.0	
Neurological	5 (45.5%)	2.76 (0.72–10.52)	0.128	3 (60%)	8.25 (0.80–85.56)	0.058
OBGYN	5 (26.3%)	1.18 (0.36–3.90)	0.784	1 (20.0%)	1.38 (0.10–19.64)	0.814
Orthopedic	2 (12.5%)	0.47 (0.10–2.36)	0.352	0 (0.0%)	0.85 (0.67–1.07)	0.551
Otolaryngology	7 (36.8%)	1.93 (0.63–5.91)	0.246	4 (57.1%)	7.33 (0.88–61.33)	0.052
Plastic	7 (13.5%)	0.52 (0.19–1.41)	0.192	0 (0.0%)	0.85 (0.67–1.07)	0.274
Urology	1 (10.0%)	0.37 (0.04–3.18)	0.346	0 (0.0%)	0.85 (0.67–1.07)	0.672
**Formaldehyde**						
*Academic level*						
Attending	17 (23.6%)	1.0		2 (11.8%)	1.0	
Resident/fellow	15 (13.5%)	0.51 (0.23–1.09)	0.082	6 (40.0%)	5.00 (0.83–33.33)	0.066
*Surgical specialty*						
General	10 (17.9%)	1.0		1 (10.0%)	1.0	
Neurological	6 (54.5%)	5.52 (1.40–21.72)	**0.009**	3 (50.0%)	9.00 (0.66–122.79)	0.074
OBGYN	2 (10.5%)	0.54 (0.11–2.73)	0.457	0 (0.0%)	0.90 (0.73–1.11)	0.640
Orthopedic	0 (0.0%)	0.13 (0.01–2.42)	0.174	-	-	-
Otolaryngology	4 (21.1%)	1.23 (0.34–4.49)	0.757	0 (0.0%)	0.90 (0.73–1.11)	0.512
Plastic	9 (17.3%)	0.96 (0.36–2.60)	0.940	4 (44.4%)	7.20 (0.62–83.34)	0.089
Urology	1 (10.0%)	0.51 (0.06–4.50)	0.539	0 (0.0%)	0.90 (0.73–1.11)	0.740
**Methylmethacrylate**						
*Academic level*						
Attending	17 (23.6%)	1.0		0 (0.0%)	1.0	
Resident/fellow	5 (4.5%)	0.15 (0.05–0.43)	**<0.001**	2 (40.0%)	25.00 (0.97–642.23)	0.052
*Surgical specialty*						
General	1 (1.8%)	1.0		0 (0.0%)	1.0	
Neurological	6 (54.5%)	66.00 (6.57–662.57)	**<0.001**	1 (16.7%)	1.20 (0.84–1.716)	0.659
OBGYN	1 (5.3%)	3.06 (0.18–51.39)	0.416	0 (0.0%)	-	-
Orthopedic	7 (43.8%)	42.78 (4.69–390.20)	**<0.001**	0 (0.0%)	-	-
Otolaryngology	1 (5.3%)	3.06 (0.18–51.39)	0.416	0 (0.0%)	-	--
Plastic	6 (11.5%)	7.17 (0.83–61.77)	0.073	1 (16.7%)	1.20 (0.84–1.72)	0.659
Urology	0 (0.0%)	0.98 (0.95–1.02)	0.670	-	-	-
**Surgical scrub**						
*Academic level*						
Attending	39 (54.2%)	1.0		13 (33.3%)	1.0	
Resident/fellow	51 (45.9%)	1.38 (0.77–2.50)	0.277	13 (25.5%)	0.68 (0.27–1.69)	0.416
*Surgical specialty*						
General	27 (48.2%)	1.0		7 (25.9%)	1.0	
Neurological	8 (72.7%)	2.86 (0.69–11.93)	0.137	4 (50.0%)	2.86 (0.56–14.60)	0.198
OBGYN	9 (47.4%)	0.97 (0.34–2.74)	0.949	1 (11.1%)	0.36 (0.04–3.39)	0.355
Orthopedic	9 (56.3%)	1.38 (0.45–4.23)	0.571	2 (22.2%)	0.82 (0.14–4.90)	0.824
Otolaryngology	9 (47.4%)	0.97 (0.34–2.74)	0.949	2 (22.2%)	0.82 (0.14–4.90)	0.824
Plastic	22 (42.3%)	0.79 (0.37–1.68)	0.538	8 (36.4%)	1.63 (0.48–5.546)	0.430
Urology	6 (60.0%)	1.61 (0.41–6.34)	0.492	2 (33.3%)	1.429 (0.21–9.56)	0.712

**Table 3:** Receipt and frequency of formal training for physical occupational hazards stratified by academic level and surgical specialty.

Characteristic	Receipt of formal training			Annual (or more frequent) training		
	No. (%)	OR (95% CI)	*p*-value	No. (%)	OR (95% CI)	*p*-value
**Ergonomics**						
*Academic level*						
Attending	28 (38.9%)	1.0		6 (21.4%)	1.0	
Resident/fellow	37 (33.3%)	0.79 (0.42–1.45)	0.443	12 (32.4%)	1.75 (0.56–5.56)	0.326
*Surgical specialty*						
General	18 (32.1%)	1.0		6 (33.3%)	1.0	
Neurological	5 (45.5%)	1.76 (0.47–6.54)	0.395	2 (40%)	1.33 (0.17–10.25)	0.782
OBGYN	7 (36.8%)	1.23 (0.42–3.66)	0.707	2 (28.6%)	0.80 (0.12–5.40)	0.819
Orthopedic	5 (31.3%)	0.96 (0.29–3.18)	0.946	0	0.67 (0.48–0.92)	0.133
Otolaryngology	10 (52.6%)	2.35 (0.81–6.78)	0.111	2 (20%)	0.50 (0.80–3.13)	0.454
Plastic	19 (36.5%)	1.22 (0.55–2.69)	0.631	5 (26.3%)	0.71 (0.17–2.94)	0.641
Urology	1 (10.0%)	0.24 (0.03–2.00)	0.154	1 (100%)	0.33 (0.173–0.64)	0.179
**Patient Lifting**						
*Academic level*						
Attending	30 (41.7%)	1.0		5 (16.7%)	1.0	
Resident/fellow	26 (23.4%)	0.43 (0.23–0.81)	**0.009**	8 (30.8%)	2.22 (0.63–7.69)	0.213
*Surgical specialty*						
General	19 (33.9%)	1.0		6 (31.6%)	1.0	
Neurological	5 (45.5%)	1.62 (0.44–6.01)	0.466	2 (40.0%)	1.44 (0.19–11.04)	0.722
OBGYN	6 (31.6%)	0.90 (0.30–2.74)	0.851	0 (0.0%)	0.68 (0.50–0.93)	0.114
Orthopedic	4 (25%)	0.65 (0.18–2.29)	0.499	0 (0.0%)	0.68 (0.50–0.93)	0.191
Otolaryngology	7 (36.8%)	1.14 (0.38–3.36)	0.818	1 (14.3%)	0.361 (0.04–3.70)	0.378
Plastic	13 (25%)	0.65 (0.28–1.50)	0.310	4 (30.8%)	0.96 (0.21–4.42)	0.961
Urology	2 (20%)	0.49 (0.09–2.52)	0.384	0 (0.0%)	0.68 (0.50–0.93)	0.347
**Prolonged Standing**						
*Academic level*						
Attending	7 (9.7%)	1.0		2 (28.6%)	1.0	
Resident/fellow	6 (5.4%)	0.53 (0.17–1.64)	0.273	4 (66.7%)	5.00 (0.47–50.00)	0.170
*Surgical specialty*						
General	3 (5.4%)	1.0		2 (66.7%)	1.0	
Neurological	2 (18.2%)	3.93 (0.57–26.88)	0.139	1 (50.0%)	0.50 (0.01–19.56)	0.709
OBGYN	1 (5.3%)	0.98 (0.10–10.04)	0.987	0 (0.0%)	0.33 (0.07–1.65)	0.248
Orthopedic	0 (0.0%)	0.946 (0.89–1.01)	0.344	-	-	-
Otolaryngology	2 (10.5%)	2.08 (0.32–13.50)	0.435	1 (50.0%)	0.50 (0.01–19.56)	0.709
Plastic	5 (9.6%)	1.88 (0.43–8.29)	0.399	2 (40.0%)	0.33 (0.02–6.65)	0.465
Urology	0 (0.0%)	0.95 (0.89–1.01)	0.454	-	-	-
**Radiation**						
*Academic level*						
Attending	55 (76.4%)	1.0		22 (40.0%)	1.0	
Resident/fellow	63 (56.8%)	0.40 (0.21–0.79)	**0.007**	25 (39.7%)	0.99 (0.47–2.08)	0.972
*Surgical specialty*						
General	37 (66.1%)	1.0		13 (35.1%)	1.0	
Neurological	8 (72.7%)	1.37 (0.33–5.77)	0.667	5 (62.5%)	3.08 (0.63–14.98)	0.152
OBGYN	9 (47.4%)	0.46 (0.16–1.33)	0.148	2 (22.2%)	0.53 (0.10–2.92)	0.459
Orthopedic	13 (81.3%	2.23 (0.56–8.77)	0.245	5 (38.5%)	1.15 (0.31–4.26)	0.830
Otolaryngology	14 (73.7%)	1.44 (0.45–4.59)	0.539	6 (42.9%)	1.385 (0.40–4.86)	0.611
Plastic	29 (55.8%)	0.65 (0.30–1.41)	0.272	14 (48.3%)	1.72 (0.68–4.65)	0.281
Urology	8 (80.0%)	2.05 (0.40–10.65)	0.384	2 (25.0%)	0.62 (0.11–3.50)	0.581
**Surgical Noise**						
*Academic level*						
Attending	10 (13.9%)	1.0		3 (30.0%)	1.0	
Resident/fellow	13 (11.7%)	1.22 (0.50–2.94)	0.664	4 (30.8%)	1.04 (0.17–6.25)	0.968
*Surgical specialty*						
General	5 (8.9%)	1.0		1 (20.0%)	1.0	
Neurological	5 (45.5%)	8.50 (1.90–31.12)	**0.002**	2 (40.0%)	2.67 (0.16–45.14)	0.490
OBGYN	1 (5.3%)	0.57 (0.06–5.18)	0.611	0 (0.0%)	0.80 (0.52–1.24)	0.624
Orthopedic	0 (0.0%)	0.91 (0.84–0.99)	0.215	-	-	-
Otolaryngology	2 (10.5%)	1.20 (0.21–6.76)	0.836	1 (50.0%)	4.00 (0.12–136.96)	0.427
Plastic	9 (17.3%)	2.14 (0.67–6.85)	0.195	3 (33.3%)	2.00 (0.15–26.73)	0.597
Urology	1 (10.0%)	1.13 (0.12–10.87)	0.914	0 (0.0%)	0.80 (0.52–1.24)	0.624
**Surgical Smoke**						
*Academic level*						
Attending	27 (37.5%)	1.0		7 (25.9%)	1.0	
Resident/fellow	22 (19.8%)	0.41 (0.21–0.80)	**0.008**	10 (45.5%)	2.38 (0.71–7.69)	0.153
*Surgical specialty*						
General	13 (23.2%)	1.0		5 (38.5%)	1.0	
Neurological	6 (54.5%)	3.97 (1.04–15.15)	**0.035**	2 (33.3%)	0.80 (0.11–6.10)	0.829
OBGYN	2 (10.5%)	0.39 (0.08–1.91)	0.232	0 (0.0%)	0.62 (0.40–0.95)	0.283
Orthopedic	5 (31.3%)	1.50 (0.44–5.12)	0.513	0 (0.0%)	0.62 (0.40–0.95)	0.103
Otolaryngology	9 (47.4%)	3.12 (1.05–9.28)	**0.041**	3 (33.3%)	0.80 (0.14–4.75)	0.806
Plastic	13 (25.0%)	1.10 (0.46–2.67)	0.828	7 (53.8%)	1.87 (0.39–8.89)	0.431
Urology	1 (10.0%)	0.37 (0.43–3.18)	0.346	0 (0.0%)	0.62 (0.40–0.95)	0.439

#### Chemical hazards

Surgical trainees were less likely to have been trained in cytotoxic drugs (OR 0.22, *p*<0.001) and methylmethacrylate (OR 0.15, *p*<0.001) than attending surgeons. Across surgical specialties, Neurosurgeons (OR 66.00, *p*<0.001) and Orthopedic surgeons (OR 42.78, *p*<0.001) were also found to have greater odds of receiving training in methylmethacrylate. Neurosurgeons were more likely to be trained in formaldehyde (OR 5.52, *p* = 0.009) (Table 2).

#### Physical hazards

Surgical trainees had lower odds of receiving training in patient lifting (OR 0.43, *p* = 0.009), radiation (OR 0.40, *p* = 0.007), and surgical smoke (OR 0.41, *p* = 0.041) than attending surgeons. Otolaryngologists (OR 3.12, *p* = 0.041) and Neurosurgeons (OR 3.97, *p* = 0.035) were also found to have greater odds of receiving training in surgical smoke. Neurosurgeons were more likely to be trained in surgical noise (OR 3.97, *p* = 0.035) (Table 3).

### Frequency of occupational hazard training

We then analyzed the influence of academic level and surgical specialty on frequency of training (Tables 1–3). Bloodborne pathogens and needlestick or sharp injuries were the most frequently trained; more than half of the trained respondents received training at yearly intervals (57%, and 55%, respectively). Frequency of occupational hazard training was similar across academic levels. However, Orthopedic surgeons were less likely to receive training in anesthetic gases more than once (OR 0.17, *p* = 0.047**)** (Table 2).

### Satisfaction with occupational hazard training

Overall, respondents who received education believed that they had satisfactory training on a majority of occupational hazards (S3 Table). Training on bloodborne pathogens and needlestick or sharps injury training was found to be “very good” (median rating 4, IQR: 3–5). On further analysis, satisfaction with training differed significantly for two chemical hazards. Trainees had a higher median satisfaction score for formaldehyde training than attendings (4 vs 3, *p* = 0.037). Across surgical specialties, no statistical difference in satisfaction was noted for any occupational hazard training.

### Occupational hazard exposure

Respondents also reported their frequency of exposure to each occupational hazard within the last year (S4 Table). Bloodborne pathogens, surgical smoke, patient lifting, surgical noise, prolonged standing, and surgical scrub were reported to have the highest frequency of exposure (Daily). Respondents reported rarely experiencing exposure to methylmethacrylate, needlestick/sharp injuries, cytotoxic drugs, and formaldehyde (Yearly). Subgroup analysis showed different patterns of hazard exposure for attendings and trainees. Furthermore, the odds of exposure to occupational hazards was not equal across surgical specialties (Table 4).

**Table 4:** Median frequency of exposure and odds of increased exposure to occupational operating room hazards stratified by academic level and surgical specialty.

	Median exposure frequency	Odds Ratio (95% Confidence Interval)	*p-value*
**Bloodborne pathogens**			
*Academic level*			
Attending	Few times per week	1.0	
Resident/fellow	Daily	5.26 (2.70–9.86)	**<0.001**
*Surgical specialty*			
General	Daily		
Neurological	Daily	0.86 (0.21–3.45)	0.838
OBGYN	Daily/Few times per week	0.66 (0.22–1.96)	0.454
Orthopedic	Few times per week	0.35 (0.11–1.06)	0.064
Otolaryngology	Few times per week	0.55 (0.19–1.57)	0.265
Plastic	Daily	0.62 (0.27–1.42)	0.262
Urology	Daily	1.87 (0.35–9.96)	0.463
**Needlestick/Sharps injury**			
*Academic level*			
Attending	Yearly	1.0	
Resident/fellow	Yearly	0.63 (0.36–1.12)	0.116
*Surgical specialty*			
General	Yearly	1.0	
Neurological	Yearly	1.01 (0.30–3.39)	0.987
OBGYN	Yearly	0.93 (0.35–2.55)	0.902
Orthopedic	Yearly	1.23 (0.44–3.47)	0.684
Otolaryngology	Yearly	0.32 (0.11–0.93)	**0.036**
Plastic	Monthly	1.80 (0.89–3.65)	0.101
Urology	Yearly	0.97 (0.27–3.43)	0.962
**Cytotoxic drugs**			
*Academic level*			
Attending	Yearly	1.0	
Resident/fellow	Yearly	3.03 (1.69–5.55)	**<0.001**
*Surgical specialty*			
General	Yearly	1.0	
Neurological	Yearly	1.54 (0.48–5.00)	0.471
OBGYN	Yearly	0.40 (0.14–1.09)	0.074
Orthopedic	Never	0.13 (0.04–0.42)	**0.001**
Otolaryngology	Yearly	0.86 (0.33–2.25)	0.759
Plastic	Yearly	0.96 (0.48–1.93)	0.920
Urology	Yearly/Monthly	3.15 (0.93–10.64)	0.064
**Formaldehyde**			
*Academic level*			
Attending	Yearly	1.0	
Resident/fellow	Yearly	2.08 (1.18–3.70)	**0.011**
*Surgical specialty*			
General	Yearly	1.0	
Neurological	Yearly	1.23 (0.36–4.20)	0.730
OBGYN	Yearly	1.82 (0.91–3.63)	0.088
Orthopedic	Yearly	0.61 (0.22–1.70)	0.342
Otolaryngology	Yearly	1.19 (0.45–3.13)	0.731
Plastic	Monthly	0.76 (0.23–2.50)	0.655
Urology	Yearly	1.64 (0.64–4.22)	0.308
**Methylmethacrylate**			
*Academic level*			
Attending	Yearly	1.0	
Resident/fellow	Yearly	2.86 (1.59–5.05)	**<0.001**
*Surgical specialty*			
General	Yearly	1.0	
Neurological	Monthly	8.64 (2.60–28.70)	**<0.001**
OBGYN	Never/Yearly	0.55 (0.20–1.55)	0.260
Orthopedic	Weekly	29.02 (9.60–87.71)	**<0.001**
Otolaryngology	Yearly	2.27 (0.86–6.00)	0.096
Plastic	Monthly	4.10 (1.98–8.47)	**<0.001**
Urology	Yearly	0.78 (0.22–2.79)	0.705
**Surgical scrub**			
*Academic level*			
Attending	Few times per week	1.0	
Resident/fellow	Daily	8.79 (4.51–17.15)	**<0.001**
*Surgical specialty*			
General	Daily	1.0	
Neurological	Daily	1.43 (0.36–5.77)	0.612
OBGYN	Few times per week	0.76 (0.27–2.20)	0.618
Orthopedic	Few times per week	0.63 (0.21–1.90)	0.420
Otolaryngology	Few times per week	0.63 (0.23–1.77)	0.389
Plastic	Daily	1.93 (0.80–4.64)	0.143
Urology	Daily	1.24 (0.29–5.25)	0.772
**Patient lifting**			
*Academic level*			
Attending	Weekly	1.0	
Resident/fellow	Daily	19.79 (9.62–40.69)	**<0.001**
*Surgical specialty*			
General	Daily	1.0	
Neurological	Daily	2.27 (0.53–9.65)	0.265
OBGYN	Few times per week/Daily	1.69 (0.56–5.15)	0.353
Orthopedic	Few times per week	0.88 (0.28–2.76)	0.835
Otolaryngology	Few times per week	0.84 (0.30–2.36)	0.747
Plastic	Daily	0.68 (0.31–1.49)	0.333
Urology	Daily	1.44 (0.33–6.17)	0.620
**Prolonged standing**			
*Academic level*			
Attending	Few times per week	1.0	
Resident/fellow	Daily	11.78 (5.70–24.34)	**<0.001**
*Surgical specialty*			
General	Daily	1.0	
Neurological	Daily	1.98 (0.39–10.14)	0.412
OBGYN	Few times per week	0.23 (0.08–0.67)	**0.007**
Orthopedic	Daily	1.62 (0.39–6.65)	0.503
Otolaryngology	Few times per week	0.48 (0.16–1.44)	0.194
Plastic	Daily	0.76 (0.31–1.89)	0.556
Urology	Daily	0.33 (0.08–1.36)	0.125
**Radiation**			
*Academic level*			
Attending	Monthly	1.0	
Resident/fellow	Weekly	5.14 (2.85–9.27)	**<0.001**
*Surgical specialty*			
General	Weekly	1.0	
Neurological	Few times per week	4.59 (1.39–15.13)	**0.012**
OBGYN	Yearly	0.18 (0.07–0.49)	**0.001**
Orthopedic	Few times per week	6.93 (2.40–19.94)	**<0.001**
Otolaryngology	Monthly	0.55 (0.21–1.40)	0.210
Plastic	Weekly	0.99 (0.50–1.95)	0.972
Urology	Few times per week	5.56 (1.59–19.53)	**0.007**
**Surgical noise**			
*Academic level*			
Attending	Few times per week	1.0	
Resident/fellow	Daily	10.87 (5.51–21.43)	**<0.001**
*Surgical specialty*			
General	Daily	1.0	
Neurological	Daily	1.40 (0.35–5.61)	0.637
OBGYN	Few times per week	0.39 (0.14–1.09)	0.074
Orthopedic	Few times per week	0.86 (0.27–2.69)	0.790
Otolaryngology	Few times per week	0.45 (0.16–1.22)	0.117
Plastic	Daily	1.49 (0.64–3.46)	0.358
Urology	Daily	0.95 (0.23–3.83)	0.939
**Surgical smoke**			
*Academic level*			
Attending	Few times per week	1.0	
Resident/fellow	Daily	18.54 (8.71–39.53)	**<0.001**
*Surgical specialty*			
General	Daily	1.0	
Neurological	Daily	0.80 (0.18–3.52)	0.763
OBGYN	Weekly/Few times per week	0.16 (0.05–0.47)	**0.001**
Orthopedic	Few times per week	0.32 (0.097–1.07)	0.064
Otolaryngology	Few times per week	0.35 (0.11–1.06)	0.064
Plastic	Daily	0.91 (0.35–2.35)	0.848
Urology	Daily	1.17 (0.23–6.13)	0.848

#### Biological hazards

Trainees had greater odds of experiencing frequent exposure to bloodborne pathogens compared to attending surgeons (OR 5.26, *p*<0.001). Otolaryngologists reported low odds of exposure to needlestick/sharp injuries (OR 0.32, *p* = 0.036).

#### Chemical hazards

Trainees reported increased odds of exposure to methylmethacrylate (OR 2.86, *p*<0.001), cytotoxic drugs (OR 3.03, *p*<0.001), and formaldehyde (2.08, *p* = 0.011) than attending surgeons. Orthopedic surgeons reported lower odds of exposure to cytotoxic drugs (OR 0.13, *p* = 0.001). The odds of methylmethacrylate exposure was significantly greater among Plastic surgery (OR 1.80, *p*<0.001), Neurosurgery (OR 8.64, *p*<0.001), and Orthopedic surgery (OR 29.02, *p*<0.001).

#### Physical hazards

Compared to attendings, residents and fellows were more likely to be exposed to patient lifting (OR 19.79, *p*<0.001), radiation (OR 5.14, *p*<0.001), prolonged standing (OR 11.78, *p*<0.001), surgical noise (OR 10.87, *p*<0.001), and surgical smoke (OR 18.54, *p*<0.001) when compared to surgical trainees. Neurosurgery (OR 4.59, *p* = 0.012), orthopedic surgery (OR 6.93, *p*<0.001), and urology (OR 5.56, *p* = 0.007) had increased odds of radiation exposure while OBGYN had lower odds of encountering radiation (OR 0.18, *p* = 0.001). Conversely, exposure to surgical smoke (OR 0.16, *p* = 0.001) and prolonged standing (OR 0.23, *p* = 0.007) was less likely among OBGYN physicians.

We then assessed the relationship between receipt of training and exposure frequency for each occupational hazard. Surgeons who received training on methylmethacrylate had greater odds of exposure (OR 3.03, *p* = 0.028) compared to surgeons who did not receive training. No significant association between receipt training and exposure frequency was noted for any other occupational hazard.

### Multivariable analysis

We adjusted for academic level, surgical specialty, and receipt of hazard training in a multivariate ordinal regression analysis. We found that residents and fellows still experienced greater odds of exposure to bloodborne pathogens (OR 5.42, *p*<0.001), cytotoxic drugs (OR 3.60, *p*<0.001), formaldehyde (OR 2.19, *p* = 0.007), methylmethacrylate (OR 3.71, *p*<0.001), surgical scrub (OR 8.76, *p*<0.001), prolonged standing (OR 12.18, *p*<0.001), patient lifting (OR 20.49, *p*<0.001), radiation (OR 5.21, *p*<0.001), surgical noise (OR 11.02, *p*<0.001), and surgical smoke (OR 19.69, *p*<0.001) compared to attendings. Whereas odds of exposure to needlestick or sharps injury remained similar between surgical trainees and attending surgeons (OR 0.64, *p* = 0.120).

## Discussion

Occupational safety and efficient workflow depend largely on the OR infrastructure, comfort level of the operative environment, recognition and awareness of potential hazards that are inescapable and part of the profession. Our study demonstrates a defining difference between specialties and an influence of training level on exposure and education. Training is not universal for any of the occupational hazards assessed. Despite a higher exposure frequency to various occupational hazards, surgical trainees were less likely to receive formal hazard training than attendings. Furthermore, surgical trainees had a greater exposure frequency to occupational hazards. Our results suggest that occupational hazard training may be selectively allocated to certain surgical specialties based on predicted frequency of hazard exposure.

### Training and frequency

The Occupational Safety and Health Administration (OSHA) has created resources that provide information and training on hospital-wide hazards however healthcare worker training is limited to traditional hazards such as bloodborne pathogens and needlestick/sharp injuries [1, 2]. While surgeons are mandated to complete an OSHA biological hazards training, no such requirement exists for nontraditional hazards found in the OR. A worrisome finding among our respondents was the lack of global training on occupational hazards; no hazard had a 100% training rate. Furthermore, traditional occupational hazards (bloodborne pathogens and needlestick/sharp injuries) demonstrated the highest frequency of training at an annual rate whereas nontraditional hazard training often demonstrated a rate of only once in a surgeon’s career. Receiving training once per career may not result in retention of knowledge. Studies demonstrate that regular repetition of training is needed to retain high quality knowledge in medicine [15, 16]. This discrepancy may be due to the widespread acceptance of biological hazards as a hospital-wide harm [17–20]. In addition, a lack of or a more lenient implementation of institutional policy may exist for the nontraditional hazards that have little to no clinical data to support their impact on surgeon health. Nonetheless, previous studies have shown that nontraditional occupational hazards can be detrimental [4–6, 21–24]. Interestingly, attending surgeons and surgical trainees were disproportionately trained in occupational hazards. A reason for this difference may be that attending surgeons possess a greater opportunity for experience through their involvement at conferences/panels, CME courses, hospital affiliations, and department specific education. Across surgical specialties, hazards training demonstrated a specialty specific pattern. Surgical specialties such as Neurosurgery and Orthopedic surgery were found to be trained significantly more in hazards such as methylmethacrylate or surgical smoke. Radiation and cytotoxic drugs showed no significant pattern. Although specialty-specific, this may mean that no specialty is receiving more or less training. Overall, the complacency of the training received is an indication that surgeons may not understand the implications of exposure to nontraditional hazards.

### Exposure

Respondents reported an expected daily exposure to a majority of the occupational hazards. The higher frequency of exposure among surgical trainees is noteworthy. Surgical trainees had a greater frequency of exposure than attending surgeons, which is attributed to the nature of residency. Residents spend, on average, 80 hours per week in the hospital. In that time, they manage a caseload of several senior surgeons and operate daily/nightly. On the other hand, attendings were less frequently exposed owing to the fact that they have dedicated operative and clinic days, and thus spend less time in the OR.

Surgical specialty-dependent exposure patterns were observed among several sub-specialties. Neurosurgery, Orthopedic surgery, and Urology shared a higher exposure frequency to radiation while Plastic surgery, Neurosurgery, and Orthopedic surgery were found to have a greater exposure to methylmethacrylate. Alternatively, exposure to radiation, surgical smoke, and prolonged standing was less likely among OBGYN. These exposure patterns may be viewed as predictable given the nature of operations performed by certain specialties. Neurosurgeons, Urologists, and Orthopedic surgeons have higher user rates of intraoperative imaging; methylmethacrylate has extensive use in arthroplasties and cranioplasties [25]. Interestingly, no statistical significance in exposure was found across specialties that perform a wide range of procedures (such as General surgery) or serve as consultants. Thus, the exposure experienced during consults appears to be incidental and underestimated.

### Exposure and training

Fundamental to this survey was the assessment of training on hazard exposure. Although not uniformly distributed, our data suggests that surgical trainees reported frequent exposure to bloodborne pathogens, radiation, methylmethacrylate, prolonged standing, patient lifting, surgical smoke, cytotoxic drugs, formaldehyde, and surgical noise than attending surgeons that was irrespective of formal training, demonstrating increased vulnerability in the setting of a knowledge gap. Several factors that may contribute to this knowledge gap include poor personal protective equipment (PPE) compliance, inconsistent reporting protocols, and unfamiliarity with OSHA safety guidelines. Despite this, a lack of training to those more vulnerable to occupational hazards may have disastrous consequences (e.g. infertility in residents who are in prime fertile years, sickness resulting in medical leave from training and delays until graduation). Previous studies have shown the impact that resident education has on decreasing hazard exposure [26]. Resident education at one institution demonstrated a significant decrease in mini-C arm time when performing fracture reduction compared with radiation exposure prior to receiving the education [26, 27]. Given the grave consequences of ongoing exposure to many of these hazards, it is imperative that more stringent and regular training is implemented.

### Limitations

Our study has several limitations. First, is the difference between our respondent and non-respondent populations. Our results suggest that trainees and certain surgical specialties were more likely to self-select and complete the survey. This introduces a bias and caution must be taken when extrapolating these results to a greater population. Secondly, the survey is based on self-report. All self-reported surveys are limited by the honesty of the respondents and accurate interpretation of survey questions. Efforts were made to ensure confidentiality of the responses to minimize this potential bias. In addition, recall bias with survey and acquiescence bias with Likert scales may overestimate or underestimate satisfaction. Thirdly, a survey response rate of 33.1% may limit generalizability of the results; however, this rate is comparable to the response rates found in other surveys involving surgeons [28–30]. The small sample size, particularly in subgroup analysis, may influence the results. On one hand, surgeons truly concerned about occupational exposures may have been more likely to respond than surgeons uninterested in hazards. Thirdly, responses from a multi-institutional hospital system that shares one department per specialty may have an impact on the external validity of the results. Lastly, receipt of formal training is only one method to receive information on occupational hazards. It is possible that surgeons learn about occupational hazards in a less structured format, such as word of mouth or idle talk in the OR. Future studies investigating surgeon knowledge of hazards are warranted.

## Conclusion

We highlight gaps between training, perceived importance, and actual practice of occupational risk management among surgeons. In addition to this, supportive data that demonstrates the link between hazard and illness in this population is limited. We encourage medical institutions and surgical specialties to educate the next generation of surgeons on occupational hazards and ensure their protection during training–for the sake of surgeon safety.

## Supporting information

S1 Table Demographic characteristics of survey respondents and non-respondents. (DOCX)

S2 Table Occupational hazards ranked from most trained to least trained. (DOCX)

S3 Table Median rating of training received on occupational hazards based on a 5-point Likert scale (1 –inadequate, 5 excellent). (DOCX)

S4 Table Median frequency of exposure to occupational hazards. (DOCX)

S1 File Occupational hazards Qualtrics survey. (PDF)
