# Supplementary material for: Occupational exposures in the operating room: Are surgeons well-equipped?
Source: PLoS One. 2021 Jul 2;16(7):e0253785. doi: 10.1371/journal.pone.0253785 (PMC8253435; doi:10.1371/journal.pone.0253785)
Supplement: S1 Table — (DOCX) [file pone.0253785.s001.docx]

| **S1 Table.** Demographic characteristics of survey respondents and non-respondents. | | | |
| --- | --- | --- | --- |
| **Characteristic** | **Respondents** | **Non-Respondents** | ***p*-value** |
| Total | 183 | 370 |  |
| *Gender* |  |  | **<0.001** |
| Female | 97 (53.1%) | 125 (33.6%) |  |
| Male | 84 (45.9%) | 247 (66.4%) |  |
| Non-binary | 2 (1.1%) | - |  |
| *Age* |  |  |  |
| 25-34 | 104 (56.8%) | - |  |
| 35-44 | 46 (25.1%) | - |  |
| 45-54 | 22 (12.0%) | - |  |
| 55-64 | 8 (4.4%) | - |  |
| 65+ | 3 (1.6%) | - |  |
| *Race* |  |  |  |
| Asian | 28 (15.3%) | - |  |
| Black/African descent | 14 (7.7%) | - |  |
| Caucasian | 111 (60.7%) | - |  |
| Middle Eastern | 7 (3.8%) | - |  |
| Multi-racial | 10 (5.5%) | - |  |
| Prefer not to say | 13 (7.1%) | - |  |
| *Identify as Hispanic/Latino?* |  |  |  |
| Yes | 16 (8.7%) | - |  |
| No | 167 (91.3%) | - |  |
| ***Academic level*** |  |  |  |
| PGY1 | 15 (8.2%) | 43 (11.6%) | 0.275 |
| PGY2 | 12 (6.6%) | 32 (8.7%) | 0.493 |
| PGY3 | 21 (11.5%) | 27 (7.3%) | 0.138 |
| PGY4 | 25 (13.7%) | 19 (5.1%) | **<0.001** |
| PGY5 | 8 (4.4%) | 20 (5.4%) | 0.752 |
| PGY6 | 10 (5.5%) | 4 (1.1%) | **0.005** |
| PGY7 | 7 (3.8%) | 4 (1.1%) | 0.064 |
| PGY8+ | 2 (1.1%) | 2 (0.5%) | 0.842 |
| Fellow | 11 (6.0%) | 15 (4.1%) | 0.417 |
| Academic Surgeon | 72 (39.3%) | 204 (55.1%) | **<0.001** |
| *Surgical specialty* |  |  |  |
| General Surgery | 56 (30.6%) | 85 (23.0%) | 0.067 |
| Neurosurgery | 11 (6.0%) | 47 (12.7%) | **0.023** |
| OBGYN | 19 (10.4%) | 84 (22.7%) | **<0.001** |
| Orthopedic Surgery | 16 (8.7%) | 54 (14.6%) | 0.070 |
| Otolaryngology (ENT) | 19 (10.4%) | 58 (15.7%) | 0.118 |
| Plastic & Reconstructive Surgery | 52 (28.4%) | 9 (2.4%) | **<0.001** |
| Urology | 10 (5.5%) | 33 (8.9%) | 0.209 |
| *PGY,* postgraduate year*; OBGYN,* Obstetrics & Gynecology, *ENT,* Ear, Nose, and Throat. **Bold text** denotes statistical significance. | | | |
