# Supplementary material for: Occupational exposures in the operating room: Are surgeons well-equipped?
Source: PLoS One. 2021 Jul 2;16(7):e0253785. doi: 10.1371/journal.pone.0253785 (PMC8253435; doi:10.1371/journal.pone.0253785)
Supplement: S2 Table — (DOCX) [file pone.0253785.s002.docx]

| **S2 Table.** Occupational hazards ranked from most trained to least trained. | | |
| --- | --- | --- |
| **Rank** | **Occupational Hazard** | **Study respondents (*n*=183)** |
| 1 | Bloodborne pathogens | 176 (96.2%) |
| 1 | Needlestick/Sharp injuries | 176 (96.2%) |
| 3 | Radiation | 118 (64.5%) |
| 3 | Methylmethacrylate | 118 (64.5%) |
| 5 | Surgical scrub | 90 (49.2%) |
| 6 | Ergonomics | 65 (35.5%) |
| 7 | Patient lifting | 56 (30.6%) |
| 8 | Surgical smoke | 49 (26.8%) |
| 8 | Anesthetic gases | 49 (26.8%) |
| 10 | Cytotoxic drugs | 40 (21.9%) |
| 11 | Formaldehyde | 32 (17.5%) |
| 12 | Surgical noise | 23 (12.6%) |
| 13 | Prolonged standing | 13 (7.1%) |
