# Supplementary material for: Occupational exposures in the operating room: Are surgeons well-equipped?
Source: PLoS One. 2021 Jul 2;16(7):e0253785. doi: 10.1371/journal.pone.0253785 (PMC8253435; doi:10.1371/journal.pone.0253785)
Supplement: S3 Table — (DOCX) [file pone.0253785.s003.docx]

| **S3 Table.** Median rating of training received on occupational hazards based on a 5-point Likert scale (1 – inadequate, 5 excellent) | |
| --- | --- |
| **Occupational Hazard** | **Median rating (Q1, Q3)** |
| Bloodborne pathogens | 4 (3, 5) |
| Sharp injuries | 4 (3, 5) |
| Prolonged standing | 3 (3, 5) |
| Methylmethacrylate | 3 (3, 4.25) |
| Anesthetic gases | 3 (3, 4) |
| Cytotoxic drugs | 3 (3, 4) |
| Ergonomics | 3 (3, 4) |
| Formaldehyde | 3 (3, 4) |
| Patient lifting | 3 (3, 4) |
| Radiation | 3 (3, 4) |
| Surgical scrub | 3 (3, 4) |
| Surgical smoke | 3 (3, 4) |
| Surgical noise | 3 (2, 4) |
| *Q1*, lower quartile; *Q3*, upper quartile | |
