# Supplementary material for: Occupational exposures in the operating room: Are surgeons well-equipped?
Source: PLoS One. 2021 Jul 2;16(7):e0253785. doi: 10.1371/journal.pone.0253785 (PMC8253435; doi:10.1371/journal.pone.0253785)
Supplement: S4 Table — (DOCX) [file pone.0253785.s004.docx]

| **S4 Table.** Median frequency of exposure to occupational hazards | |
| --- | --- |
| **Occupational Hazard** | **Median exposure frequency** |
| Bloodborne pathogens | Daily |
| Surgical smoke | Daily |
| Patient Lifting | Daily |
| Prolonged standing | Daily |
| Surgical Noise | Daily |
| Surgical scrub | Daily |
| Radiation | Weekly |
| Sharp injuries | Yearly |
| Methylmethacrylate | Yearly |
| Cytotoxic drugs | Yearly |
| Formaldehyde | Yearly |
