# Supplementary material for: Occupational exposures in the operating room: Are surgeons well-equipped?
Source: PLoS One. 2021 Jul 2;16(7):e0253785. doi: 10.1371/journal.pone.0253785 (PMC8253435; doi:10.1371/journal.pone.0253785)
Supplement: S1 File — (PDF) [file pone.0253785.s005.pdf]

## Default Question Block

What is your current level of training or position?

- ○ PGY 1
- ○ PGY 2
- ○ PGY 3
- ○ PGY 4
- ○ PGY 5
- ○ PGY 6
- ○ PGY 7
- ○ PGY 8 +
- ○ Fellow
- ○ Academic Surgeon
- ○ Private Surgeon

What is your specialty?

- ☐ Cardiothoracic Surgery
- ☐ General Surgery
- ☐ Neurosurgery
- ☐ OBGYN
- ☐ Oral and Maxillofacial Surgery
- ☐ Orthopedic Surgery

- ☐ Otolaryngology (ENT)
- ☐ Pediatric Surgery
- ☐ Plastic & Reconstructive Surgery
- ☐ Trauma Surgery
- ☐ Urology
- ☐ Vascular Surgery
- ☐ ______ Other

What is your current age?

- ○ 18-24
- ○ 25-34
- ○ 35-44
- ○ 45-54
- ○ 55-64
- ○ 65+

What is your race/ethnicity?

- ○ American Indian/Alaska Native
- ○ Asian/Asian descent
- ○ Black/African descent
- ○ White/Caucasian/European descent
- ○ Native Hawaiian/Pacific Islander
- ○ Middle Eastern/ North African descent
- ○ ______ Multi-racial
- ○ ______ Other
- ○ Prefer not to say

Do you identify as Hispanic/Latino?

- Yes
- No

Please indicate your gender identity:

- Cis-Female
- Transgender Female
- Cis-Male
- Transgender Male
- Non-binary
- ____________ Prefer to describe:

Have you ***ever*** received formal training or supplemental information on the following occupational exposures/hazards:

| | Yes | No |
|---|---|---|
| Ergonomics (Equipment use) | ○ | ○ |
| Bloodborne Pathogens | ○ | ○ |
| Patient Lifting | ○ | ○ |
| Formaldehyde | ○ | ○ |
| Surgical Smoke (Bovie, Laser, etc) | ○ | ○ |
| Radiation Exposure | ○ | ○ |

| | | |
|---|---|---|
| Methylmethacrylate | ○ | ○ |
| Surgical Noise/Suctioning/Vibratory devices) | ○ | ○ |
| Needle-stick/Sharp Injuries | ○ | ○ |
| Prolonged Standing (>3 hrs) | ○ | ○ |
| Povidone-Iodine vs Chlorhexidine scrub | ○ | ○ |
| Anesthetic Gases | ○ | ○ |
| Handling of Cytotoxic drugs (HIPEC, Mitomycin) | ○ | ○ |

How adequate was your training or supplemental information on the following?

| | Excellent | Very Good | Satisfactory | Very Poor | Unacceptable |
|---|---|---|---|---|---|
| Ergonomics (Equipment use) | ○ | ○ | ○ | ○ | ○ |
| Bloodborne Pathogens | ○ | ○ | ○ | ○ | ○ |
| Patient Lifting | ○ | ○ | ○ | ○ | ○ |
| Formaldehyde | ○ | ○ | ○ | ○ | ○ |
| Surgical Smoke (Bovie, Laser, etc) | ○ | ○ | ○ | ○ | ○ |
| Radiation Exposure | ○ | ○ | ○ | ○ | ○ |
| Methylmethacrylate | ○ | ○ | ○ | ○ | ○ |
| Surgical Noise | ○ | ○ | ○ | ○ | ○ |

| | | | | | |
|---|---|---|---|---|---|
| Needle-stick/Sharp Injuries | ○ | ○ | ○ | ○ | ○ |
| Prolonged Standing (>3 hrs) | ○ | ○ | ○ | ○ | ○ |
| Povidone-Iodine vs. Chlorhexidine Scrub | ○ | ○ | ○ | ○ | ○ |
| Anesthetic Gases | ○ | ○ | ○ | ○ | ○ |
| Handling of cytotoxic drugs (HIPEC, Mitomycin) | ○ | ○ | ○ | ○ | ○ |

How often are you trained on the following?

| | Weekly | Monthly | Yearly | Only < 2 times |
|---|---|---|---|---|
| Ergonomics (Equipment use) | ○ | ○ | ○ | ○ |
| Bloodborne Pathogens | ○ | ○ | ○ | ○ |
| Patient Lifting | ○ | ○ | ○ | ○ |
| Formaldehyde | ○ | ○ | ○ | ○ |
| Surgical Smoke (Bovie, Laser, etc) | ○ | ○ | ○ | ○ |
| Radiation Exposure | ○ | ○ | ○ | ○ |
| Methylmethacrylate | ○ | ○ | ○ | ○ |
| Surgical Noise | ○ | ○ | ○ | ○ |
| Needle-stick/Sharp Injuries | ○ | ○ | ○ | ○ |

| | | | | |
|---|---|---|---|---|
| Prolonged Standing (>3 hrs) | ○ | ○ | ○ | ○ |
| Povidone-Iodine vs. Chlorhexidine Scrub | ○ | ○ | ○ | ○ |
| Anesthetic Gases | ○ | ○ | ○ | ○ |
| Handling of cytotoxic drugs (HIPEC, Mitomycin) | ○ | ○ | ○ | ○ |

Please rate the extent to which the following items may be hazardous:

| | Definitely | Probably | Possibly | Probably Not | Definitely Not |
|---|---|---|---|---|---|
| Prolonged Standing (standing > 3hrs) | ○ | ○ | ○ | ○ | ○ |
| Bovie/Laser Smoke | ○ | ○ | ○ | ○ | ○ |
| Methylmethacrylate | ○ | ○ | ○ | ○ | ○ |
| Ergonomics (Equipment use) | ○ | ○ | ○ | ○ | ○ |
| Entering a room with contact precautions | ○ | ○ | ○ | ○ | ○ |
| Radiation | ○ | ○ | ○ | ○ | ○ |
| Anesthetic Gases | ○ | ○ | ○ | ○ | ○ |
| Formaldehyde | ○ | ○ | ○ | ○ | ○ |
| Surgical Noise | ○ | ○ | ○ | ○ | ○ |

| | | | | | |
|---|---|---|---|---|---|
| Chlorhexidine hand scrub | ○ | ○ | ○ | ○ | ○ |
| Povidone Iodine hand scrub | ○ | ○ | ○ | ○ | ○ |
| Handling of Cytotoxic drugs (Mitomycin, HIPEC) | ○ | ○ | ○ | ○ | ○ |

Please rate the extent to which the following items may be hazardous to a pregnant surgeon:

| | Definitely | Probably | Possibly | Probably Not | Definitely Not |
|---|---|---|---|---|---|
| Prolonged Standing (> 3hrs) | ○ | ○ | ○ | ○ | ○ |
| Bovie/Laser Smoke | ○ | ○ | ○ | ○ | ○ |
| Methylmethacrylate | ○ | ○ | ○ | ○ | ○ |
| Ergonomics (Equipment use) | ○ | ○ | ○ | ○ | ○ |
| Bedroom with contact precautions | ○ | ○ | ○ | ○ | ○ |
| Radiation | ○ | ○ | ○ | ○ | ○ |
| Anesthetic Gases | ○ | ○ | ○ | ○ | ○ |
| Formaldehyde | ○ | ○ | ○ | ○ | ○ |
| Surgical Noise | ○ | ○ | ○ | ○ | ○ |
| Chlorhexidine hand scrub | ○ | ○ | ○ | ○ | ○ |

| | | | | | |
|---|---|---|---|---|---|
| Povidone Iodine hand scrub | ○ | ○ | ○ | ○ | ○ |
| Handling of Cytotoxic drugs (Mitomycin, HIPEC) | ○ | ○ | ○ | ○ | ○ |
| Tissue debridement of infected tissue | ○ | ○ | ○ | ○ | ○ |

Please rate the frequency at which you are exposed to the following:

| | **Regularly** (daily) | **Often** (Few times a week) | **Sometimes** (once/week) | **Occasionally** (Monthly) | **Rarely** (Yearly) | Nev |
|---|---|---|---|---|---|---|
| **Prolonged Standing** (>3 hours) | ○ | ○ | ○ | ○ | ○ | ○ |
| **Blood Borne Pathogens** (Contact precaution rooms, Patient fluid products, etc) | ○ | ○ | ○ | ○ | ○ | ○ |
| **Patient Lifting** (transferring, etc) | ○ | ○ | ○ | ○ | ○ | ○ |
| **Formaldehyde** | ○ | ○ | ○ | ○ | ○ | ○ |
| **Surgical Smoke** (Bovie, Laser, etc) | ○ | ○ | ○ | ○ | ○ | ○ |
| **Radiation Exposure** | ○ | ○ | ○ | ○ | ○ | ○ |
| **Methylmethacrylate** | ○ | ○ | ○ | ○ | ○ | ○ |

| | | | | | | |
|---|---|---|---|---|---|---|
| **Surgical Noise** (Vibratory devices, Suction devices, etc) | ○ | ○ | ○ | ○ | ○ | ○ |
| **Needle-stick/Sharps injury** | ○ | ○ | ○ | ○ | ○ | ○ |
| **Surgical Hand Scrub** (Povidone/Iodine) | ○ | ○ | ○ | ○ | ○ | ○ |
| **Anti-Neoplastic drugs** (HIPEC, mitomycin) | ○ | ○ | ○ | ○ | ○ | ○ |

Have you ever been pregnant during any portion of your surgical training or surgical career?

- Yes
- No

Have occupational hazards or exposures ever influenced your decision to become pregnant?

- Yes
- No

While pregnant, how often did you:

| | **Regularly** (daily) | **Often** (Few times a week) | **Sometimes** (once/week) | **Occasionally** (Monthly) | **Rare** (Yearl |
|---|---|---|---|---|---|

| | | | | | |
|---|---|---|---|---|---|
| Participate in surgical cases > 3 hours? | ○ | ○ | ○ | ○ | ○ |
| Lift patients | ○ | ○ | ○ | ○ | ○ |
| Attend Cadaver lab | ○ | ○ | ○ | ○ | ○ |
| Enter a room with contact precautions (MRSA, etc) | ○ | ○ | ○ | ○ | ○ |
| Not use a smoke evacuation system during a surgical case | ○ | ○ | ○ | ○ | ○ |
| Participate in cases with radiation exposure | ○ | ○ | ○ | ○ | ○ |
| Participate in cases that used methylmethacrylate | ○ | ○ | ○ | ○ | ○ |
| Experience a needle-stick/sharp injury | ○ | ○ | ○ | ○ | ○ |
| Participate in a case with anti-neoplastic drugs (HIPEC, mitomycin, etc) | ○ | ○ | ○ | ○ | ○ |
| Use Povidone/Iodine to scrub | ○ | ○ | ○ | ○ | ○ |
| Participate in a case that involved debriding infected tissue | ○ | ○ | ○ | ○ | ○ |

Please indicate which personal protective equipment you wore while pregnant for the following scenarios (select all that apply):

| | Surgical Mask | Surgical Mask with Eyeshield | N95 Mask | Surgical Gown | Lead Apron | Yellow Isolation Gown | Steril Glove |
|---|---|---|---|---|---|---|---|
| Cadaver Lab | ☐ | ☐ | ☐ | ☐ | ☐ | ☐ | ☐ |

| | | | | | | | |
|---|---|---|---|---|---|---|---|
| Patient Room with Contact Precautions | ☐ | ☐ | ☐ | ☐ | ☐ | ☐ | ☐ |
| Surgical case - any | ☐ | ☐ | ☐ | ☐ | ☐ | ☐ | ☐ |
| Surgical case - tissue debridement | ☐ | ☐ | ☐ | ☐ | ☐ | ☐ | ☐ |
| Surgical case - methylmethacrylate use | ☐ | ☐ | ☐ | ☐ | ☐ | ☐ | ☐ |
| Surgical case - radiation exposure | ☐ | ☐ | ☐ | ☐ | ☐ | ☐ | ☐ |
| Surgical case - Direct blood splatter | ☐ | ☐ | ☐ | ☐ | ☐ | ☐ | ☐ |
| Surgical case - Laser | ☐ | ☐ | ☐ | ☐ | ☐ | ☐ | ☐ |
| Emergency room - patient room | ☐ | ☐ | ☐ | ☐ | ☐ | ☐ | ☐ |

Please indicate which personal protective equipment you wear for the following scenarios (select all that apply):

| | Surgical Mask | Surgical Mask with Eyeshield | N95 mask | Surgical Gown | Lead apron | Yellow Isolation Gown | Sterile Glove |
|---|---|---|---|---|---|---|---|
| Cadaver Lab | ☐ | ☐ | ☐ | ☐ | ☐ | ☐ | ☐ |
| Patient Room with Contact Precautions | ☐ | ☐ | ☐ | ☐ | ☐ | ☐ | ☐ |
| Surgical case - any | ☐ | ☐ | ☐ | ☐ | ☐ | ☐ | ☐ |

| | | | | | | | |
|---|---|---|---|---|---|---|---|
| Surgical case - tissue debridement | ☐ | ☐ | ☐ | ☐ | ☐ | ☐ | ☐ |
| Surgical case - methylmethacrylate use | ☐ | ☐ | ☐ | ☐ | ☐ | ☐ | ☐ |
| Surgical case - radiation exposure | ☐ | ☐ | ☐ | ☐ | ☐ | ☐ | ☐ |
| Surgical case - direct blood splatter | ☐ | ☐ | ☐ | ☐ | ☐ | ☐ | ☐ |
| Surgical case - Laser | ☐ | ☐ | ☐ | ☐ | ☐ | ☐ | ☐ |
| Emergency room - patient room | ☐ | ☐ | ☐ | ☐ | ☐ | ☐ | ☐ |

Did you experience pregnancy complications while working in the hospital (you may choose more than one)?

- ☐ No
- ☐ Preterm birth
- ☐ Cesarean section
- ☐ Low birth weight
- ☐ Spontaneous abortion (miscarriage)
- ☐ ________ Other

Did you report any exposures during your pregnancy?

- ○ Yes
- ○ No

What exposure did you report?

Are there any additional occupational hazards that are notable to mention?

Powered by Qualtrics
